# Assessing ChatGPT Ability to Answer Frequently Asked Questions About Essential Tremor

**DOI:** 10.5334/tohm.917

**Published:** 2024-07-03

**Authors:** Cristiano Sorrentino, Vincenzo Canoro, Maria Russo, Caterina Giordano, Paolo Barone, Roberto Erro

**Affiliations:** 1 Department of Medicine, Surgery and Dentistry “Scuola Medica Salernitana”, Neuroscience Section, University of Salerno, Via Allende 43, 84081 Baronissi, SA, Italy; 2 Department of Neurology, “Umberto I”Hospital, Nocera Inferiore (SA), Italy

**Keywords:** Essential tremor, Movement disorders, Large language Model, Artificial intelligence, ChatGPT

## Abstract

**Background:**

Large-language models (LLMs) driven by artificial intelligence allow people to engage in direct conversations about their health. The accuracy and readability of the answers provided by ChatGPT, the most famous LLM, about Essential Tremor (ET), one of the commonest movement disorders, have not yet been evaluated.

**Methods:**

Answers given by ChatGPT to 10 questions about ET were evaluated by 5 professionals and 15 laypeople with a score ranging from 1 (poor) to 5 (excellent) in terms of clarity, relevance, accuracy (only for professionals), comprehensiveness, and overall value of the response. We further calculated the readability of the answers.

**Results:**

ChatGPT answers received relatively positive evaluations, with median scores ranging between 4 and 5, by both groups and independently from the type of question. However, there was only moderate agreement between raters, especially in the group of professionals. Moreover, readability levels were poor for all examined answers.

**Discussion:**

ChatGPT provided relatively accurate and relevant answers, with some variability as judged by the group of professionals suggesting that the degree of literacy about ET has influenced the ratings and, indirectly, that the quality of information provided in clinical practice is also variable. Moreover, the readability of the answer provided by ChatGPT was found to be poor. LLMs will likely play a significant role in the future; therefore, health-related content generated by these tools should be monitored.

## Introduction

Essential Tremor (ET) is defined as a syndrome of action tremor of the upper limbs which can further involve other body regions, in the absence of additional overt neurological signs and of at least 3 years duration [1,2,3]. ET is one of the most frequent movement disorders with an overall prevalence estimate of about 1% which significantly increases with age, affecting more than 6% of people aged 65 years or older [4]. Despite its high prevalence, ET remains frequently undiagnosed or misdiagnosed [5], its public recognition being low. For instance, in a survey of both neurological patients and caregivers attending general neurology, vascular, or movement disorders clinics, only about 10–30% of respondents reported awareness of the condition [6]. Other studies have demonstrated that ET is frequently misdiagnosed, mainly owing to symptom heterogeneity and ambiguity [5,6,7]. Moreover, in a survey including 2864 respondents, it was shown that clinical care of ET is provided by the family physician in up to 26% of cases and only 19% of patients had seen a movement disorder specialist [8], which might suggest that the information about the condition that are received by the patients could be of variable quality and accuracy.

The challenges associated with the diagnosis of ET, its poor recognition and the possible variability about the information received by patients in different clinical settings might theoretically be one of reasons driving online, health-related, information seeking behaviors. In a previous study on movement disorders, we in fact demonstrated that most of the queries on Google were related to definitions, causes and symptoms of different conditions, being possibly conducted to aid self-diagnosis [8]. Furthermore, another infodemiologic study carried out in the field of movement disorders has shown that the term “tremor” was the most queried on Google, with the relative search volume of “Essential Tremor” being 4 times higher than that of “Tourette’s syndrome” [10].

The practice of using the internet to access real-time information on health-related issues is pervasively increasing worldwide, but studies have shown inherent potential risks associated with accessing poor quality, incorrect and/or difficult to read information [11,12], which might in turn lead to negative health behaviors and outcomes [13]. Internet and social media are helpful tools to increase awareness about a particular condition, but there is also the risk of perpetuating myths and disinformation [14].

ChatGPT, introduced by OpenAI in November 2022 [15], is one of the available large language models (LLMs) tools using computational artificial intelligence (AI) that are specifically trained to process and generate text. It enables users to engage in human-like conversations and provides detailed responses about any topic, including healthcare, and is increasingly replacing common search engines. It took only 5 days for ChatGPT’s user base to reach one million following the launch of GPT-3.5 in November 2022 and, according to the latest available data, ChatGPT currently has around 180.5 million users with an average of about 13 million unique visitors that had used it per day in January 2024, more than double the levels of December 2023 [16]. The potential value of LLMs has been already shown in several health-care areas. For instance, in 2023 Singhal and colleagues described Med-PaLM, a LLM designed to provide high quality and accurate answers to medical questions, which demonstrated an accuracy of 67% answering a dataset consisting of US Medical Licensing Exam-style questions, surpassing the prior state of the art by more than 17% [17]. Moreover, Lim and colleagues showed that LLM chatbots, including ChatGPT and Google Bard, provided comprehensive responses to myopia-related health concerns [18], while Goodman and colleagues showed that GPT-3.5 and GPT-4 were largely accurate for addressing complex medical queries across 17 different medical specialties [19].

However, LLMs knowledge derives from a broad range of information, including books, articles, websites, and other written material available online, some of which is likely to be inaccurate or, at least, difficult to understand by laypeople, as shown in a recent work on cardiopulmonary resuscitation [20]. Therefore, also in view of the potential larger use of LLMs tools in the near future, we aimed to analyze the accuracy, relevance, comprehensiveness and overall perceived value of answers provided by ChatGPT to a list of frequently asked questions (FAQs) about ET. We selected ChatGPT over LLMs because of its popularity, public accessibility, convenient usability, and human-like output. The latter is achieved through an incorporated reward model based on human feedback, known as reinforcement learning from human feedback, which results in more credible output than other LLMs [21,22].

## Methods

FAQs about ET were first produced by one of the authors (RE) who, based on his clinical experience with ET, developed a list of 25- most received - questions revolving around diagnosis, clinical aspects of ET, differential diagnosis, therapeutic options including alternative treatments and progression of the condition. This list was proposed to three ET patients [2 males and 1 female, aged 67, 59 and 63 years, with a disease duration of 41, 35, and 39 years, respectively, and with a phenotype of “pure” ET with tremor in the upper limbs only of mild to moderate severity (TETRAS performance sub-scale score of 24, 14, and 18, respectively)], who were asked to independently rank them according to their perceived importance. Rankings were subsequently averaged (e.g., [(rank position × number of responses for each rank position)/total number of responses] and the top 10 questions used for the current study. They were subsequently ordered according to the type of question (e.g. first 2 questions about the condition more in general, followed by 4 about its progression, followed by 4 about possible treatments).

On December 20th, 2023, we asked ChatGPT (using the free version 3.5) to provide answers using each question as input. The questions were entered sequentially in a single interaction with ChatGPT (e.g., in a single navigating session), the model was not primed with any other question or statement and no question was repeated twice.

The generated answers were then evaluated by two groups: 5 professionals (e.g., group 1) and 15 laypeople (group 2) who included people who were never formally exposed to information about ET recruited among friends/family members of the professionals who participated in the study.

Group 1 included one neurology resident (CG), three junior neurologists (CS, VC, MR) and one early career faculty (RE), all participating in clinical and research activities about ET.

Participants were asked to evaluate each ChatGPT-generated answer with a score ranging from 1 to 5 (e.g., 1 = poor; 2 = not satisfactory; 3 = adequate; 4 = good; 5 = excellent) according to 5 parameters: clarity (e.g. How well is the answer written, structured and presented?), relevance (e.g., How well does it answer the question that was asked? A relevant answer addresses the question directly), accuracy [(only for professionals); e.g. ‘How correct it is’ - is it factually correct and free from errors?], comprehensiveness (e.g. Does the answer include all or nearly all the information you would expect?), and overall value of the response, in line with a previous study on cardiopulmonary resuscitation [20]. All participants but one (RE) were blinded to the fact that answers were generated using ChatGPT and were told they would have been used for an informational leaflet for patients. Beyond descriptive statistics, ratings were compared between groups (professionals vs laypeople) using the Mann-Whitney test to explore whether literacy about ET could influence the ratings and are presented as median with interquartile ranges (IQR), p < 0.05 being deemed as significant. We further calculated percentage agreement of ratings for each parameter for each question in the two groups, the level of agreement being deemed good when ≥75% of raters expressed a vote equal to 4 or 5 for any item [23].

Finally, the readability of ChatGPT answers was evaluated by means of several parameters, using an on-line software (readable.com). Namely, we used the Flesch Reading Ease test [24], which was initially developed for school books and uses two core measures (e.g. word length and sentence length), higher scores indicating the material is easier to read; the Simple Measure of Gobbledygook (SMOG) index [25], which is widely used to check for health-related content and estimates the years of education a person needs to understand a piece of writing based on the number of polysyllables (words of 3 or more syllables) in a fixed number of sentences; the Coleman-Liau Index [26] that, unlike syllable-based readability indices, relies on word-length in characters, its output approximating the U.S. grade level thought necessary to comprehend the text; and the FORCAST Grade Level [27], which relies on the number of “easy” words with one syllable in a sample of 100–150 words and provides a value that estimates the number of years of education a reader requires to understand a text. We also used two further measures that the on-line software (e.g. readable.com) provides by combining the aforementioned readability metrics. Namely, the “Readability Score” that uses an A-E rating system (the text aimed at the general public should be grade B or better) and the Reach metric, which represents the percentage of reached audience among the general literate population.

## Results

The two groups did not differ in terms of age [32 (6) vs 42 (32) years, professionals vs laypeople, respectively, z = 27.5; p = 0.395] or sex distribution (60% vs 46.7% male, professionals vs laypeople, respectively, x^2^ = 0.267; p = 0.500); however, education was significantly higher in professionals than in laypeople [19 (3) vs 18 (2), respectively, z = 27.5; p < 0.001).

Overall, all answers were rated relatively good, with median scores ranging between 4 and 5, by both professionals and laypeople, although with wide IQRs ranging from 1 to 4. Answers received similar scores by both type of raters independently from the type of question (e.g. about clinical aspects, progression, therapeutic options). Complete aggregate results of the ratings, presented by type of rater, can be found in Table 1.

**Table 1:** Question comparison between professional healthcare and laypeople.


QUESTION	QUALITY/CLARITY [MEDIAN, (IQR)]			RELEVANCE [MEDIAN, (IQR)]			ACCURACY [MEDIAN, (IQR)]	COMPREHENSIVENESS [MEDIAN, (IQR)]			OVERALL JUDGEMENT [MEDIAN, (IQR)]		
			
	PROFESSIONAL (N = 5)	LAYPEOPLE (N = 15)	p	PROFESSIONAL (N = 5)	LAYPEOPLE (N = 15)	p	PROFESSIONAL (N = 5)	PROFESSIONAL (N = 5)	LAYPEOPLE (N = 15)	p	PROFESSIONAL (N = 5)	LAYPEOPLE (N = 15)	p

**What is essential tremor?**	[5.0 (2.0)]	[4.0 (1.0)]	0.866	[5.0 (1.0)]	[5.0 (1.0)]	0.866	[5.0 (2.0)]	[5.0 (2.0)]	[5.0 (1.0)]	0.800	[4.0 (2.0)]	[4.0 (1.0)]	1.000

**Is essential tremor like Parkinson’s disease?**	[4.0 (2.0)]	[4.0 (1.0)]	0.230	[5.0 (3.0)]	[4.0 (1.0)]	0.933	[4.0 (3.0)]	[4.0 (2.0)]	[4.0 (1.0)]	0.266	[4.0 (3.0)]	[4.0 (1.0)]	0.497

**I received a diagnosis of essential tremor. Will I get worse?**	[5.0 (2.0)]	[4.0 (1.0)]	0.933	[5.0 (3.0)]	[4.0 (1.0)]	0.800	[5.0 (3.0)]	[4.0 (3.0)]	[5.0 (1.0)]	0.266	[5.0 (2.0)]	[5.0 (1.0)]	0.800

**Can I do something to slow its progression?**	[5.0 (1.0)]	[5.0 (1.0)]	0.933	[5.0 (1.0)]	[4.0 (3.0)]	0.197	[5.0 (3.0)]	[4.0 (3.0)]	[5.0 (0.0)]	0.119	[5.0 (3.0)]	[5.0 (0.0)]	0.395

**I had a diagnosis of essential tremor. I am at risk of developing the disease Parkinson’s?**	[4.0 (2.0)]	[4.0 (1.0)]	0.672	[5.0 (3.0)]	[4.0 (1.0)]	0.800	[5.0 (3.0)]	[4.0 (4.0)]	[4.0 (1.0)]	0.612	[5.0 (3.0)]	[4.0 (1.0)]	0.933

**Will I have memory problems?**	[5.0 (1.0)]	[4.0 (1.0)]	0.349	[4.0 (2.0)]	[4.0 (2.0)]	1.000	[4.0 (3.0)]	[4.0 (3.0)]	[4.0 (1.0)]	0.445	[4.0 (3.0)]	[4.0 (2.0)]	0.800

**Drugs for essential tremor aren’t working. I can take something else?**	[5.0 (1.0)]	[5.0 (1.0)]	0.933	[5.0 (2.0)]	[4.0 (1.0)]	0.800	[5.0 (3.0)]	[5.0 (3.0)]	[5.0 (1.0)]	0.612	[5.0 (3.0)]	[5.0 (1.0)]	0.553

**Can cannabis be useful for Essential Tremor?**	[5.0 (2.0)]	[4.0 (2.0)]	0.445	[4.0 (3.0)]	[4.0 (1.0)]	0.800	[4.0 (3.0)]	[4.0 (3.0)]	[4.0 (2.0)]	0.800	[4.0 (3.0)]	[4.0 (2.0)]	0.445

**I have heard of ultrasound therapy for essential tremor. Can you tell me more?**	[5.0 (1.0)]	[4.0 (1.0)]	0.266	[4.0 (1.0)]	[4.0 (1.0)]	0.800	[4.0 (2.0)]	[4.0 (2.0)]	[4.0 (1.0)]	0.933	[5.0 (2.0)]	[4.0 (2.0)]	0.800

**Does ultrasound therapy have any side effects?**	[5.0 (1.0)]	[4.0 (1.0)]	0.497	[5.0 (1.0)]	[4.0 (1.0)]	0.349	[5.0 (2.0)]	[4.0 (2.0)]	[5.0 (1.0)]	0.445	[4.0 (2.0)]	[4.0 (1.0)]	0.612


**Figure 1:**
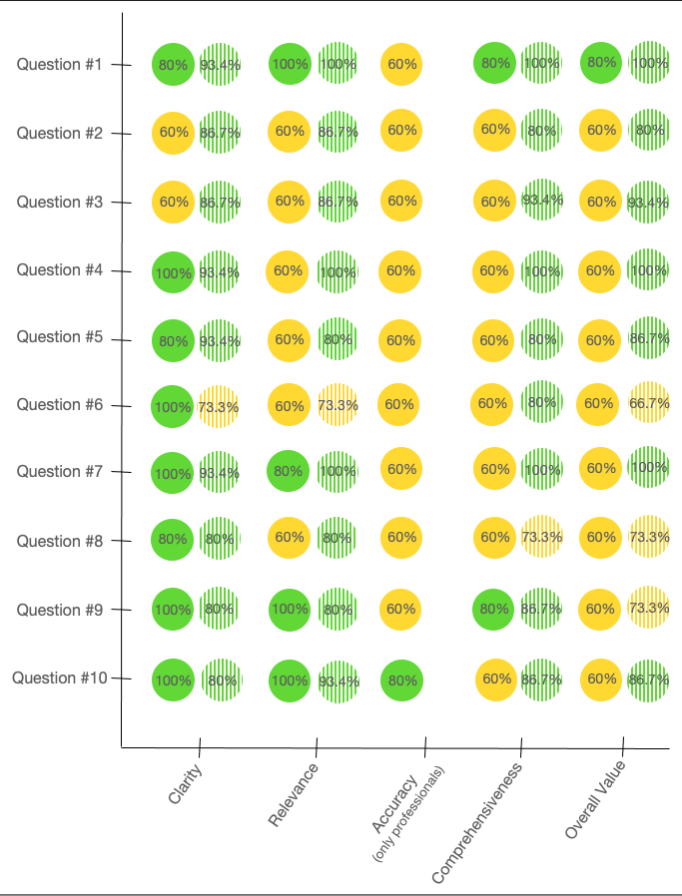
Aggregate percentage agreement of ratings ≥4 for each parameter for each question in the group of professional (plain circles) and laypeople (dashed circles). Green circles indicate good agreement (≥75%) and yellow circles moderate agreement (≥60%).

Conversely, cumulative percentage agreement of ratings ≥4 were variable between the groups. Good agreement (e.g. ≥75%) between professional raters was found for 8/10 answers in terms of clarity, 4/10 in terms of relevance, 1/10 in terms of accuracy, 2/10 in terms of comprehensiveness and 1/10 in terms of overall value of the response (Figure 1). Higher cumulative percentage agreement was found between the laypeople raters, with good scores being observed for 9/10 answers in terms of clarity, 9/10 in terms of relevance, 9/10 in terms of comprehensiveness and 7/10 in terms of overall value of the response (Figure 1). Detailed percentage agreement for each rating is available in the supplemental tables. Notably, regarding the “accuracy” parameter that was only assessed by the professionals, 7/10 answers obtained a negative rating (e.g., 2 = not satisfactory) by one or more raters (supplemental tables).

Overall readability levels were consistently poor for all questions. Details about the readability of singles answers according to different parameters are provided in Table 2.

**Table 2:** Readability metrics of the answers provided by ChatGPT.


QUESTION	FLESCH READING EASE	SMOG INDEX	COLEMAN-LIAU INDEX	FORCAST GRADE LEVEL	READABILITY SCORE	REACH

What is Essential Tremor?	32.0	15.1	13.9	12.1	D	59%

Is essential tremor like Parkinson’s disease?	28.7	14.6	14.2	12.7	D	57%

I have been diagnosed with essential tremor. Do I carry a higher risk of developing Parkinson disease?	8.1	18.5	17.2	13.2	E	25%

I have been diagnosed with ET? Will I get worse?	35.6	14.3	14.9	12.4	D	68%

Is there anything I can do to slow its progression down?	31.6	14.6	16.0	12.7	D	67%

Does ET affect memory?	13.7	17.8	17.2	13.9	E	32%

Is cannabis useful for ET?	20.0	17.0	15.2	12.4	E	42%

Prescribed drugs for essential tremor are not working. can I try something else?	20.2	17.3	17.9	13.0	E	43%

I heard about ultrasound in essential tremor. can you tell me more?	26.3	16.6	14.8	12.6	E	51%

Does ultrasound therapy have side effects?	32.6	16.0	13.7	12.1	D	57%


Considering the aggregate metrics (e.g., Readability score and Reach), 50% of the answers were graded as D and the remaining 50% as E, with the percentage of reached audience among the general literate population ranging between 25% and 68% and with 4/10 answers reaching less than 50% of the potential audience.

## Discussion

In this study we evaluated the ability of ChatGPT to answer a list of questions commonly asked by patients and their caregivers about ET. We used the version of ChatGPT that was freely available (e.g. ChatGPT-3.5) at the time of conducting the study to simulate the most likely scenario in the real world. ChatGPT was chosen over other LLMs because of its popularity. It was beyond the scope of this study to systematically assess the capabilities of ChatGPT (or other LLMs) of answering FAQs related to ET. In fact, different answers are produced each time, even from the same question, worded identically, from the same LLM. The order of questions as well as “priming” of the model (by providing for instance direction to the model such as “act as a movement disorder specialist”) might significantly affect the output. Furthermore, the output might further change in time depending on the available sources from which LLMs gather their data to generate the answers. A systematic assessment of the capabilities of LLMs to answer any queries, with their respective pros and cons, would require multiple iterations, changes in wording, different priming, etc [28,29]. Therefore, taking into account that the newly developed version 4 of ChatGPT outperforms earlier models as well as LLMs specifically fine-tuned on medical knowledge such as Med-PaLM [30], our data should be intended as providing an initial picture about the outcome of a single interaction with ChatGPT, simulating what would likely happen in the real world.

Generated answers were rated by laypeople as well as by professionals, who judged them as relatively factually correct in terms of accuracy, although with some variability. Overall, ChatGPT answers received good median scores in terms of clarity, relevance, comprehensiveness, and overall value of the response by both laypeople and professionals and with no significant difference between the type of raters. However, we also found that there was only moderate agreement between professionals in terms of relevance, comprehensiveness, accuracy and overall value of the response for most of the answers, whereas good agreement was generally observed among the laypeople. This might be explained by the different literacy about ET between the two type of raters. However, there was variable agreement even among the group of 5 professionals with most answers (7/10) receiving a negative rating (e.g 2 = not satisfactory) in terms of accuracy by at least one rater and with junior raters being more likely to give higher ratings (data not shown). The latter results might therefore indirectly indicate that the information that people receive in the real world is also of variable quality, given that ET patients are cared for by clinicians with different background, including general practitioners [8].

To best of our knowledge this is the first study trying to evaluate the answers of ChatGPT in field of movement disorders with a particular focus on ET and our results adds to a growing – although still fairly small – body of research [18,19,20,31,32,33] suggesting that ChatGPT can provide reasonably correct medical information in a human-like manner, being therefore more appreciated than static internet information, and with higher quality and more empathy than the answers provided by verified physicians [34]. However, differently from previous research on other medical discipline [18,20,31,32,33] or on multiple sclerosis [34], it should be noted that our results somehow diverge in the way that there was only moderate agreement between the professional raters. This might ultimately be related to the profound disagreement, even about movement disorder experts, about the nature of ET [35,36,37].

Contrary to previous research [20,31,32] who involved patients/care-givers affected by the specific condition being the object of the questions, we instead decided to involve laypeople who were never exposed to any information about ET, therefore avoiding any bias that might have arisen from prior knowledge about ET. The choice of selecting people never exposed to any information to ET was also driven by our previous results showing that health-related information seeking behaviors mostly occur to aid self-diagnosis prior to encounter any medical professionals [9]. We further excluded potential biases arising from any skepticism related to AI since participants were blinded to the fact that the answers were generated from a LLM.

If on the one hand side, the positive ratings especially given by the laypeople suggest that ChatGPT might be a useful tool, potentially improving patients’ engagement and reducing workload for healthcare providers, it should be also noted that readability, although the metrics we used might show intrinsic differences and were not all developed to assess medical content [38], was consistently judged very poor with a reach that was at times found as low as 25% of the potential audience. The discrepancy between the positive ratings and the poor readability scores can be easily explained by the high level of education of the subjects involved in this study. The latter should be therefore acknowledged as a limitation, considering that, for instance, according to the Flesh Reading Ease score 6/10 answers obtained a score indicating they were “extremely difficult to read” and only suitable for college graduates.

One might also argue that the list of questions was not complete to depict the complexity of the condition as we only selected the top 10 questions rated by 3 ET patients about different aspects of the condition. For instance, questions related to first-line pharmacological approaches were not included and this is reasonably due to the fact that our 3 ET patients were already treated and, therefore, might have been more likely interested in knowing about alternative options, including surgical treatments. However, although we acknowledge that the FAQs were derived from one provider and only 3 patients rather than a more robust number of each, we would not expect grossly different results by simply increasing the number of providers, patients and/or questions.

Our results show that ChatGPT provides relatively accurate, relevant, and comprehensive, yet poorly readable, answers to questions frequently asked by patients with ET. However, concerns remain around several aspects of this (and similar) LLMs. Although ChatGPT is trained on massive amounts of text data, including books, articles, and websites, it might not easily deal with disputed areas of knowledge and might not provide correct answers when contradictions are present in the input data such as in the case of ET, where profound disagreement exist even between experts [35,36,37]. Moreover, there have been reports of a tendency of the model to occasionally “hallucinate”, that is to provide confidently formulated answers with incorrect or nonsensical content [39]. Users should also be aware that ChatGPT and similar models might show bias against individuals depending on based on sex, ethnicity, or disability [40]. This issue is particularly sensitive in the field of ET, where stigma is prevalent and contribute to social dysfunction [41]. An additional concern is related to the potential for data breaches or unauthorized access to protected health information [42]. This issue should be mitigated by specific laws designed to protect the privacy and security of individuals’ health information. Finally, we evaluated ChatGPT answers in Italian and although this tool might in fact be exploited to produce text in several languages, ultimately reducing linguistic barriers [43], it is unknown whether the value including the accuracy of answers provided in different languages is similar or not.

Notwithstanding these caveats, the power of text-generating LLM tools is undeniable, and it is likely that they will be pervasively used by patients and their care-giver in the near future. While it is crucial to establish robust monitoring measures for health-related information generated by these systems, movement disorders specialists could cautiously attempt to integrate these systems into their clinical practice and make use of their great possibilities [43].

## Data Accessibility Statement

Data will be made available upon request to the corresponding author.

## Additional File

The additional file for this article can be found as follows:

10.5334/tohm.917.s1 Supplementary Tables. Detailed percentage agreement of ratings in two group of raters.
