# Supplementary material for: Assessing ChatGPT Ability to Answer Frequently Asked Questions About Essential Tremor
Source: Tremor Other Hyperkinet Mov (N Y). 2024 Jul 3;14:33. doi: 10.5334/tohm.917 (PMC11225576; doi:10.5334/tohm.917)
Supplement: Supplementary Tables. — Detailed percentage agreement of ratings in two group of raters. [file tohm-14-1-917-s1.pdf]

	Clarity			Relevance				Accuracy				Comprehensiveness					Overall value of the response			
	3	4	5	2	3	4	5	2	3	4	5	1	2	3	4	5	2	3	4	5
**Question #1**	20%	20%	60%	0%	0%	40%	60%	0%	40%	0%	60%	0%	0%	20%	20%	60%	0%	20%	40%	40%
**Question #2**	40%	40%	20%	20%	20%	0%	60%	20%	20%	20%	40%	0%	20%	20%	40%	20%	20%	20%	20%	40%
**Question #3**	40%	0%	60%	20%	20%	0%	60%	40%	0%	0%	60%	0%	40%	0%	20%	40%	40%	0%	0%	60%
**Question #4**	0%	40%	60%	40%	0%	20%	40%	40%	0%	0%	60%	0%	40%	0%	20%	40%	20%	20%	0%	60%
**Question #5**	20%	40%	40%	40%	0%	0%	60%	40%	0%	0%	60%	20%	20%	0%	20%	40%	40%	0%	0%	60%
**Question #6**	0%	40%	60%	0%	40%	20%	40%	20%	20%	20%	40%	0%	40%	0%	20%	40%	20%	20%	20%	40%
**Question #7**	0%	40%	60%	0%	20%	20%	60%	20%	20%	0%	60%	0%	20%	20%	0%	60%	20%	20%	0%	60%
**Question #8**	20%	20%	60%	20%	20%	20%	40%	20%	20%	20%	40%	0%	20%	20%	20%	40%	40%	0%	40%	20%
**Question #9**	0%	40%	60%	0%	0%	60%	40%	0%	40%	20%	40%	0%	0%	20%	40%	40%	0%	40%	0%	60%
**Question #10**	0%	40%	60%	0%	0%	40%	60%	0%	20%	20%	60%	0%	0%	40%	20%	40%	0%	40%	20%	40%

Percentage agreement of ratings in the group of professionals

	Clarity			Relevance				Comprehensiveness					Overall value of the response			
	3	4	5	2	3	4	5	1	2	3	4	5	2	3	4	5
**Question #1**	6,6%	46,7%	46,7%	0%	0%	53,6%	46,4%	0%	0%	6,6%	40%	53,4%	0%	6,6%	66,7%	26,7%
**Question #2**	20%	40%	40%	6,6%	20%	46,7%	26,7%	0%	6,6%	26,6%	26,6%	40,2%	6,6%	20%	40%	33,4%
**Question #3**	20%	40%	40%	13,3%	13,3%	53,4%	20%	0%	13,3%	6,6%	33,3%	46,8%	0%	20%	33,3%	46,7%
**Question #4**	0%	40%	60%	13,3%	0%	26,7%	60%	0%	13,3%	0%	20%	66,7%	6,6%	6,6%	20%	66,8%
**Question #5**	13,3%	46,7%	40%	13,3%	20%	26,7%	40%	6,6%	6,6%	20%	40%	26,8%	13,3%	13,3%	46,7%	26,7%
**Question #6**	26,7%	33,3%	40%	6,6%	33,3%	33,3%	26,8%	0%	13,4%	20%	33,3%	33,3%	13,3%	33,3%	20%	33,4%
**Question #7**	0%	40%	60%	0%	6,6%	60%	33,3%	0%	6,6%	6,6%	40%	46,8%	6,6%	6,6%	33,3%	53,5%
**Question #8**	26,7%	46,7%	26,6%	13,3%	20%	46,7%	20%	0%	6,6%	33,3%	40%	20,1%	26,8%	13,3%	40%	19,9%
**Question #9**	20%	46,7%	33,3%	0%	13,4%	53,3%	33,3%	0%	0%	20%	46,7%	33,3%	6,6%	33,3%	26,8%	33,3%
**Question #10**	13,3%	46,7%	40%	0%	6,6%	66,7%	26,7%	0%	0%	26,8%	26,8%	46,4%	6,6%	20%	40%	33,4%

Percentage agreement of ratings in the group of laypeople
